# Significant Lactic Acidosis from Albuterol

**DOI:** 10.5811/cpcem.2018.1.36024

**Published:** 2018-03-14

**Authors:** Maxwell Hockstein, Deborah Diercks

**Affiliations:** University of Texas Southwestern, Department of Emergency Medicine, Dallas, Texas

## Abstract

Lactic acidosis is a clinical entity that demands rapid assessment and treatment to prevent significant morbidity and mortality. With increased lactate use across many clinical scenarios, lactate values themselves cannot be interpreted apart from their appropriate clinical picture. The significance of Type B lactic acidosis is likely understated in the emergency department (ED). Given the mortality that sepsis confers, a serum lactate is an important screening study. That said, it is with extreme caution that we should interpret and react to the resultant elevated value. We report a patient with a significant lactic acidosis. Though he had a high lactate value, he did not require aggressive resuscitation. A different classification scheme for lactic acidosis that focuses on the bifurcation of the “dangerous” and “not dangerous” causes of lactic acidosis may be of benefit. In addition, this case is demonstrative of the potential overuse of lactates in the ED.

## INTRODUCTION

Given that increased lactate values (either capillary or true vascular sampling with values greater than 2mmol/L) are unequivocally associated with higher likelihoods of in-hospital mortality across many clinical scenarios (including both medical and traumatic disease), emergency physicians globally are being faced with attributing a lactate value to either a potentially devastating or not-devastating etiology. While different schematics have been presented to classify lactic acidosis, the “Type A” and “Type B” classification scheme is likely insufficient as it does not answer the question “sick or not sick?”

## CASE REPORT

We report the case of a 50-year-old male who presented to the emergency department (ED) complaining of dyspnea. The patient had a known history of asthma and felt that his symptoms were typical of his exacerbations. He noted a cough with yellow sputum but denied fevers or any pain. He also reported a history of tension headaches and hyperlipidemia; his medical history was negative for diabetes, seizures, or strokes. He had no prior surgeries. He reported taking amitriptyline 10mg as needed for headaches and was also on atorvastatin 40mg daily. He had no allergies to medications. He denied any and all alcohol intake and he was not clinically intoxicated.

The triage vital signs were as follow: blood pressure 139/105 mmHg, temperature 35.7ºC (96.3ºF), heart rate 104 beats per minute, oxygen saturation 94% on room air, and respiratory rate 20 breaths per minute. A chest radiograph did not reveal any acute abnormalities. The patient received a single dose of 15mg of albuterol, 1500mcg of ipratropium, and 60mg of prednisone shortly after his ED arrival. He remained somewhat dyspneic after this initial treatment and was given an additional 10mg of albuterol two hours later. Given that the patient required one additional treatment, the decision was made to transfer him to the observation unit for further monitoring.

As part of the admission process to the observation unit, a basic metabolic panel was ordered, which showed a sodium of 139 mmol/L, potassium of 3.2 mmol/L, chloride of 100 mmol/L, bicarbonate of 18 mmol/L, and an anion gap of 24 mmol/L. The creatinine was 0.91 mg/dL. The aspartate aminotransferase was 16 units/liter and the alanine transaminase was 22 units/liter. The white blood cell count was 7.19 x 10^9^/L. The admitting team then ordered a lactate to address the anion gap, which resulted at 9.6 mmol/L with a corresponding pH from the venous blood gas of 7.31 and a partial pressure of carbon dioxide of 34 mmHg. The lactate was repeated one hour later and resulted at 10.3 mmol/L. A standard urine drug screen, examining for amphetamines, benzodiazapines, cannabinoids, cocaine metabolites, opiates, and phencyclidine, was negative. The patient’s symptoms had improved and he remained otherwise asymptomatic.

The conundrum we faced was to determine not only whether this was a Type A or Type B lactic acidosis but also its precipitant. Given the lack of toxic appearance, hypotension, and altered mental status it was strongly felt that Type A lactic acidosis was not the culprit. The patient was admitted to our medicine service and observed. Additional albuterol treatments were withheld and the serum lactate value cleared to 1.1 mmol/L approximately 24 hours later. The patient was discharged from the hospital without incident.

## DISCUSSION

Since Huckabee’s landmark paper in 1961, which identified a group of patients with fatal lactic acidosis, lactate has been used as a test to screen for acute metabolic mismatch.^1^ Lactate is produced when pyruvate is not converted to acetyl coenzyme A (CoA) during glycolysis in the setting of normal (aerobic) cellular respiration. Contrary to popular belief, when lactate is formed from pyruvate the product exists as an anion, not with the attached proton.^2^ Therefore, it is not lactate itself that causes acidosis; rather, it is a surrogate marker for an increase in the number of protons accumulating in the failing hydrolysis of adenosine triphospate. Type A lactic acidosis is defined by the presence of shock (hypoperfusion of any tissue). Type B lactic acidosis is a term that describes any lactic acidosis not due to hypoperfusion. Therefore, the causes for Type B lactic acidosis are more varied than those for Type A lactic acidosis.

### Type A Lactic Acidosis

Clinical scenarios such as cardiac arrest, sepsis, and mesenteric ischemia are associated with severe lactic acidosis and with poor outcomes. Given the profound mortality of septic shock, checking serum lactates has been encouraged since the publication of “Surviving Sepsis”^3^ in 2012 and has been used as a screening tool for serious illness in many additional settings. In general, patients with Type A lactic acidosis require restoration of perfusion, with fluid resuscitation and/or vasopressors. In addition to these clinical pictures, seizures may cause a Type A lactic acidosis; however, our patient did not seize.^4^ He displayed no concerns for a Type A lactic acidosis, and we were directed to consider an additional precipitant.

### Type B Lactic Acidosis

Clinical entities such as drugs (therapeutic or otherwise), inborn errors of metabolism, malabsorption syndrome (responsible for elevated levels of D-Lactate, as opposed to L-Lactate) are responsible for Type B lactic acidosis. Germaine to the patient in question, the treatment team was initially perplexed as to what caused this lactate value. Causes of Type B lactic acidosis may be bifurcated as a disorder of either increased production of lactate or a decreased clearance of lactate.^5^ Increased production of lactate occurs when the rate of glycolysis increases: catecholamines, diminished pyruvate dehydrogenase activity (congenital or thiamine deficiency), malignancy, and oxidative insufficiencies (cyanide toxicity). Decreased lactate clearance occurs in hepatic enzyme inhibition, mitochondrial defects, and renal disease.

**CPC-EM Capsule**

What do we already know about this clinical entity?

Lactic acidosis has many etiologies but can be divided into Type A from hypoperfusion (e.g., sepsis) and Type B from other causes (e.g., medications such as albuterol).

What makes this presentation of disease reportable?

This lactic acid value is significantly higher than previously reported values attributed solely to albuterol in an otherwise-healthy patient.

What is the major learning point?

Though potentially indicative of serious disease, increased lactate values can also indicate a metabolic derangement not necessarily requiring resuscitation.

How might this improve emergency medicine practice?

This case demonstrates that lactic acid values should be interpreted (and treated) in the appropriate clinical context.

#### Increased Lactate Production

Catecholaminergic stimulation of the beta-2 receptor upregulates cyclic adenosine monophosphate, which in turn activates protein kinase A (PKA). PKA activation enhances glycogenolysis and yields glucose, a substrate for glycolysis. The acceleration of this process with exogenous catecholamines results in hyperlactatemia.^6^ In addition, a urine toxicology screen was checked to address sympathomimetics as they may cause an increased lactate. However, no drugs of abuse were detected. Our patient had received albuterol; however, this degree of lactate elevation from albuterol has only seldom been referenced in the literature, especially in such a well-appearing patient. Mean elevations of serum lactate after one hour of albuterol (10mg) administration in healthy volunteers have been reported to be 0.77.^7^ In known asthmatics, the mean lactate level after at least 10mg of albuterol treatment has been reported in two studies to escalate 2.94 mmol/L from normal baselines.^8^,^9^ Lactate levels in asthmatics are of particular concern because hyperlactatemia may inhibit the bronchodilatory response and that the accompanying acidosis may worsen respiratory effort.^8^

Thiamine is a cofactor in the pyruvate dehydrogenase (PDH) complex and therefore a deficiency of thiamine would decrease the activity of PDH. Decreased PDH activity would increase lactate concentrations by not catalyzing the conversion from pyruvate to acetyl-CoA. In addition to patients who chronically drink ethanol or who have beri beri, those receiving parenteral nutrition also are at risk for thiamine deficiency.^10^

The mechanisms for malignancy-driven Type B lactic acidosis are varied. The most common explanation lies in the fact that cancer cells possess high glycolytic activity, an observation termed “the Warburg effect,” initially described in 1924.^11^ This increased rate of glycolysis, as described earlier, increases serum lactate values.

D-lactate, the isomer of L-lactate, is produced mainly in patients with short-gut syndrome where colonic bacteria are exposed to larger quantities of luminal carbohydrates.^4^ D-Lactate may also be observed with propylene glycol ingestion; it has the potential to cross the blood-brain barrier and cause neurologic sequelae such as encephalopathy (slurred speech, ataxia, hallucinations, somnolence), which can last from hours to days.^12^

One of the most well-known side effects of 3-hydroxy-3-methyl-glutaryl CoA reductase inhibitors (statins) is myopathy. Infrequently, statins have been associated with increased serum lactate values of similar cardinalities. Lactic acidosis attributable to a statin is an uncommon event; rather, it is often associated with concomitant metabolic derangements.^13^,^14^ The proposed mechanism by which statins may contribute to lactic acidosis is a depletion of ubiquinone (also known as CoQ10 (an important cofactor for the electron transport chain). However, concrete evidence of a mechanism is lacking.

#### Decreased Lactate Clearance

The liver is responsible for the majority (70–75%) of lactate clearance^5^,^10^ with the remainder eliminated by the kidneys. In patients with hepatic disease or hepatic injury (e.g., ischemic hepatitis) lactate may be elevated. However, a primary insult, which would elevate the lactate in the first place, must be considered. In septic patients without septic shock, the oxygen delivery to tissues is generally increased; therefore, increased lactate levels in this subset of patients are likely due to decreased clearance rather than increased production.^5^

## CONCLUSION

The significance of Type B lactic acidosis is likely understated in the ED. Given the mortality that sepsis confers, a serum lactate is an important screening study. That said, it is with extreme caution that we should interpret and react to the resultant value. This patient, though he had a high lactate value, likely did not require aggressive resuscitation. A more rigorous classification scheme for lactic acidosis might be of clinical benefit. Emergency physicians should be aware that lactate values may be dramatically elevated for several reasons, only one of them being albuterol.

Documented patient informed consent and/or Institutional Review Board approval has been obtained and filed for publication of this case report.
